# Development of a Tetraplex Digital PCR Assay for the Detection of Invasive Snake Species in Florida, USA

**DOI:** 10.1002/ece3.70598

**Published:** 2024-11-20

**Authors:** Melissa A. Miller, Melody Bloch, Sergio A. Balaguera‐Reina, Kevin A. Olejniczak, Cynthia A. Fussell Persaud, Ericka E. Helmick, Frank J. Mazzotti, Brian W. Bahder

**Affiliations:** ^1^ Department of Wildlife Ecology and Conservation, Institute of Food and Agricultural Sciences, Fort Lauderdale Research and Education Center University of Florida Davie Florida USA; ^2^ Department of Entomology and Nematology, Institute of Food and Agricultural Sciences, Fort Lauderdale Research and Education Center University of Florida Davie Florida USA

**Keywords:** conservation, eDNA, molecular tools, monitoring, survey

## Abstract

Florida, USA is a hotspot of biological invasions with over 500 non‐native species reported. Reptiles encompass most of the non‐native wildlife with over 50 species established, many of which are sympatric and are identified as invasive due to their impacts to the environment, economy, and human health and safety. Reports of new non‐native reptiles occur, and many established non‐native reptiles continue to expand their ranges in Florida, increasing the need for multitaxa detection and monitoring capabilities. Invasive constrictor snakes are a primary focus of management efforts due to life history traits that favor successful establishment and dispersal in Florida as well as their impacts to native wildlife and Everglades restoration efforts. While traditional survey methods that rely on visual detections fail to reliably detect invasive constrictors, environmental DNA (eDNA) has proven to be a promising method for detection of cryptic and rare species across the landscape. To address emerging needs for multispecies detection and monitoring in Florida, we developed the first tetraplex dPCR assay designed for detection of four species of invasive constrictor snakes, including Burmese pythons (*Python bivittatus*), northern African pythons (*P. sebae*), boa constrictors (*Boa constrictor*), and rainbow boas (*Epicrates cenchria*). In this tetraplex assay, no cross‐amplification across species was documented. This assay serves as a valuable tool for faster and more accurate monitoring efforts of these invasive species in South Florida. Additionally, eDNA samples comprised of soil and water both tested positive for Burmese python DNA under controlled and semicontrolled conditions with DNA being detectable up to 2‐weeks post removal in soil samples. Water samples yielded positive detection as quickly as 5 min after exposure to the organism. These data highlight the utility and sensitivity of this protocol for eDNA monitoring.

## Graphical abstract

A tetraplex digital PCR assay was developed for four invasive snake species in the Florida Everglades. This assay is a resource that will be used in eDNA monitoring of these invasive species.

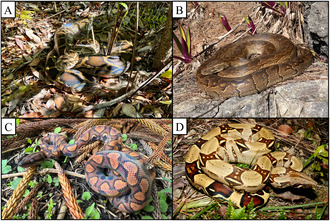

## 1 Introduction

**FIGURE 1:**
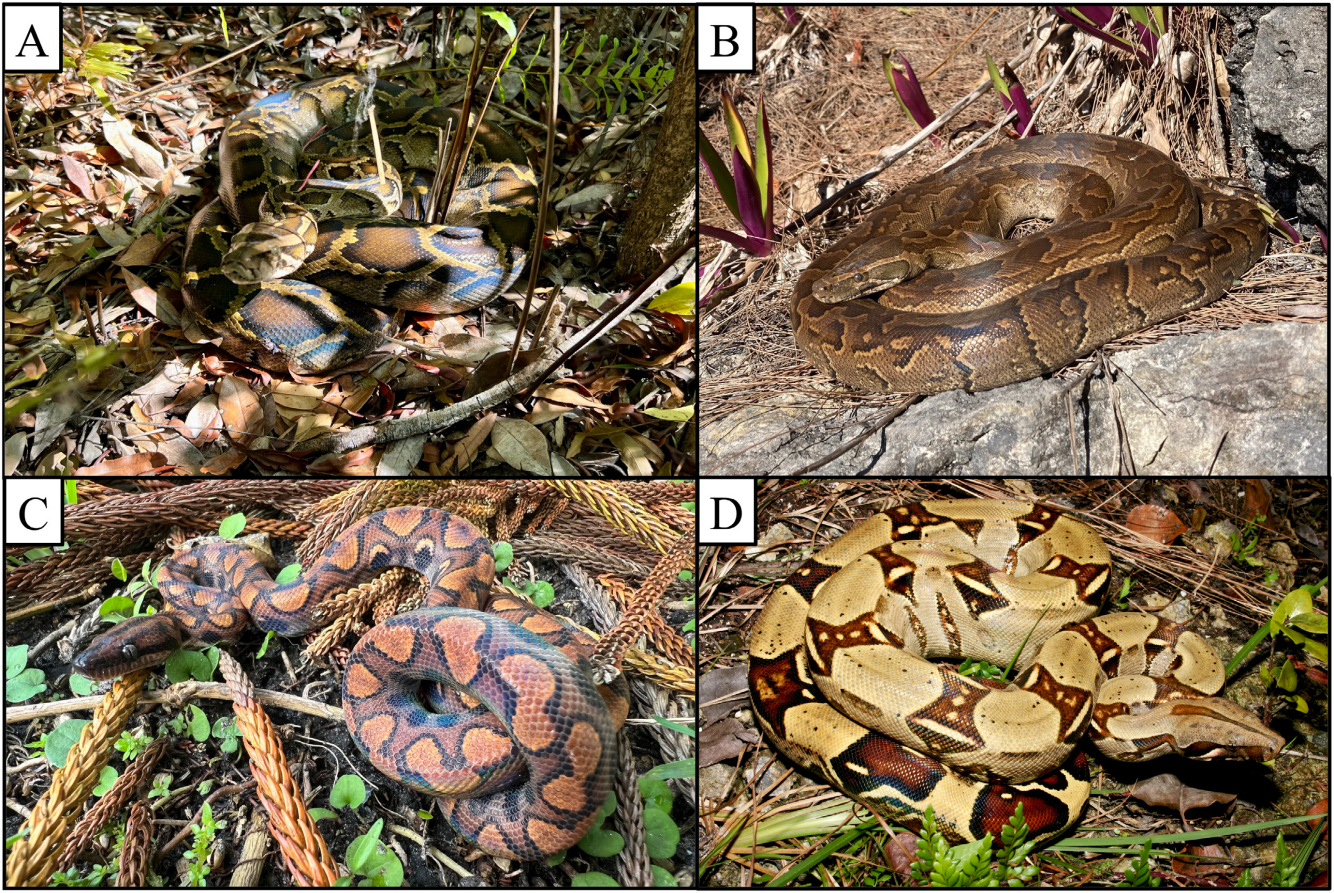
Representative specimens of the species used to design the tetraplex digital PCR assay in this study; (A) Burmese python (*P.bivittatus*), (B) northern African python (*Python sebae*), (C) rainbow boa (*E. cenchria*) and (D) boa constrictor (*B. constrictor*), Photos were provided by MAM (A, C), Anthony Flanagan (B) and Kevin Enge (D).

Florida is a hotspot of biological invasions, particularly for reptiles, due to a subtropical climate, peninsular geography, disturbed habitats, thriving exotic pet trade, and multiple major ports of entry to the United States (Engeman et al. 2011). At least 54 species of non‐native reptiles are established (i.e., breeding) in Florida, including 27% of all established non‐native reptile species known to occur globally (Capinha et al. 2017). Since establishment, many of these invasive reptiles have increased in abundance and expanded their range, with numerous species becoming sympatric. Among the most concerning are invasive large constrictor snakes, including the Burmese python (*Python bivittatus*), northern African python (*P. sebae*), and boa constrictor(*Boa constrictor*) (Figure 1) due to their large size, high reproductive rate, long life span, and ability to disperse long distances (Guzy et al. 2023). In addition, evidence supports the probable establishment of a fourth species of non‐native constrictor snake, the rainbow boa (*Epicrates cenchria*) (Figure 1), of which all size classes, including a gravid female, have been captured in the wild (MAM, pers. comm.; EDDMapS 2024). Due to known and potential impacts to native wildlife in Florida, these constrictors are considered priority species by environmental agencies (i.e., Florida Fish and Wildlife Conservation Commission and South Florida Water Management District) targeted for early detection and rapid response (EDRR) efforts, containment, or long‐term management depending on the area invaded and the time since introduction (Florida Python Control Plan 2021).

Documented impacts are known primarily for Burmese pythons, the most studied of the invasive constrictor species in Florida and include severe reductions in populations of mammalian prey species (Dorcas et al. 2012; McCleery et al. 2015), co‐introduction of invasive parasites that have spread to infect native wildlife (Miller et al. 2018, 2020), and increased prevalence of zoonotic viruses (Hoyer et al. 2017). Impacts of other invasive constrictors may yield comparable results once fully examined. Of particular concern is how these invaders may affect Everglades restoration efforts, a multi‐billion‐dollar initiative to restore the quantity, quality, timing, and distribution of water for the benefit and protection of people, habitats, and wildlife (South Florida Ecosystem Restoration Task Force 2022). To address this issue, the Invasive Exotic Species Strategic Action Framework (South Florida Ecosystem Restoration Task Force 2020) was initiated to combat the impacts of invasive species, including constrictor snakes, on these efforts as the establishment of invasive species directly threatens restoration goals.

Substantial resources have been provided to support control efforts of invasive constrictors in Florida with 10.6 million US dollars spent by federal and state agencies, and one nongovernmental organization during 2004–2021 (Florida Python Control Plan 2021) for the removal of Burmese pythons, the most abundant and widespread of these invasive snakes (EDDMapS 2024). Despite these unprecedented resources, python removal remains challenging due to their cryptic appearance and propensity for secretive behaviors and large periods of inactivity, which belies our ability to easily detect and remove this species (Guzy et al. 2023; Nafus, Mazzotti, and Reed 2020). The detection probability of Burmese pythons using data from visual encounter surveys and radio telemetry, is estimated to be < 5% (Nafus, Mazzotti, and Reed 2020) and detection likely decreases with increasing habitat complexity. In addition to low detection, Burmese pythons inhabit vast remote interior locations within the Everglades with dense vegetation and seasonal flooding, often requiring specialized equipment for human access, which can further limit the utility of visual detection methods due to logistics or lack of resources to maintain sustained monitoring efforts. A delay in detection reduces the effectiveness of rapid response and removal efforts, decreasing the likelihood of successful eradication and increasing the probability an introduced species will become established and require long‐term control. Therefore, development of methods to increase detection of invasive snakes are paramount for the success of subsequent eradication and control efforts (Hunter et al. 2015).

Environmental DNA (eDNA) sampling can aid in the detection of invasive wildlife, improve early detection, inform occupancy modeling, help determine invasion fronts, and provide managers with a method to monitor and assess eradication efforts (Hunter et al. 2015, 2019; Orzechowski et al. 2019; Morisette et al. 2021; Keller et al. 2022; Carim et al. 2019). This method has proven to be a viable tool for increasing the detection of cryptic invasive species, including Burmese pythons (Hunter et al. 2015, 2019; Orzechowski et al. 2019). Using eDNA assays and occupancy modeling, Hunter et al. (2015) estimated the detection probability of pythons to be > 91%, demonstrating the utility of eDNA for python detection compared to the < 5% detection probability of pythons using visual encounter surveys (Nafus, Mazzotti, and Reed 2020). However, despite advantages for increasing detection, particularly of secretive or rare species, eDNA has yet to be widely deployed as a detection tool for invasive species management. A contributing factor may result from a lack of confidence in eDNA detections due to the potential for false positives and false negatives, which can confound management decisions. For example, a positive eDNA detection of an invasive species that cannot be verified by other means of detection may trigger a costly response that may not be warranted (Jerde 2021). The potential for false‐negative detections may increase when environmental inhibitors, preventing the detection of DNA, are present or when samples contain trace amounts of DNA that may be undetected by the eDNA assay utilized.

Recent advances in digital PCR (dPCR) assays show promise for addressing many of these issues due to increased specificity and accuracy while allowing for multiplex testing for detection of multiple target species within a sample (Gaňová et al. 2021). Multiplex eDNA assays can be conducted rapidly and efficiently for the detection of up to six target species simultaneously with reduced resources compared to traditional eDNA assay methodologies (Gaňová et al. 2021). The development of multiplex dPCR assays is an emerging need to meet demands for early detection and monitoring of multiple target species in systems with multiple invasion events, such as South Florida. Additionally, a streamlined, automated workflow has the potential to reduce the resources required for implementing multiplex dPCR as a detection tool for large‐scale applications across the landscape, which may increase the accessibility of eDNA for use by natural resource managers for implementing monitoring programs.

Toward this goal, we developed and tested a robust multiplex assay for rapid, accurate, and cost‐effective detection of environmental DNA of multiple target species within a sample that meets the needs of natural resource managers. Specifically, our multiplex dPCR assay is designed to detect invasive constrictor snakes (Burmese python, boa constrictor, northern African python, and rainbow boa) in Florida which have substantial overlap in distribution and habitat preference. We provide a detailed account of assay development and validation on eDNA samples as well as discuss the potential advantages of this methodology for increasing the effectiveness of invasive species monitoring programs in multi‐invaded systems.

## 2 Materials and Methods

### 2.1 Animal Tissue Source and DNA Extraction

Animal tissues were harvested from specimens (northern African python, Burmese python, *Boa constrictor*, and rainbow boa) previously collected during research projects conducted by the University of Florida (UF IACUC protocol number 202200000027; FWC EXOT‐23‐63a; FWC EXOT‐23‐83) or from specimens donated by the Florida Fish and Wildlife Conservation Commission acquired through invasive species removal efforts. Euthanasia of the organisms used in this part of the study is covered by the above‐mentioned IACUC and performed according to the AVMA 2020 guidelines. Experimentation on live animals was also covered under the above‐mentioned IACUC.

Muscle tissue (100 mg) was excised from each specimen (fresh frozen collected no longer than 1 year from experiment start) and transferred to a 1.5 mL microcentrifuge tube. Total DNA was extracted using the DNeasy Blood & Tissue Kit (Qiagen) according to the manufacturer's instructions with slight modifications. In the initial lysis step, tissue was not macerated, it was left intact and allowed to lyse over a 24‐h period at 56°C. Final eluate had DNA concentration and purity measured using a NanoDropLite spectrophotometer (ThermoFisher) and was diluted to 25 ng/μL for running PCR assays.

### 2.2 PCR Assay Design

The gene and region selected for assay design was the cytochrome *c* oxidase subunit I (COI) barcoding region (5′‐half) due to high levels of variability among species (and even among populations; Kundu et al. 2020; Liu et al. 2020) allowing for easy development of species‐specific assays without cross‐amplification. Template obtained from *B. constrictor*, *E. cenchria*, *P. sebae*, and *P. bivittatus*, was screened in the initial assay using the universal COI primers LCO1490 (forward) and HCO2198 (reverse) from Folmer et al. (1994). Reactions were run in volumes of 25 μL and were comprised of 5× GoTaq Flexi Buffer (Promega, Madison, Wisconsin, USA), 25 mM MgCl2, 200 μM dNTPs, 0.5 μM of forward and reverse primer, 2% PVP‐40, 1 U GoTaq Flexi DNA polymerase (Promega, Madison, Wisconsin, USA), and 2 μL of DNA template with sterile water to increase the final reaction volume to 25 μL. Utility of these primers in snakes was previously unknown so insect DNA extract (from the planthopper *Pelitropis rotulata*) using the same protocol as that for snakes was used as a positive control and molecular grade water was used as a nontemplate control. Thermal cycling conditions were as follows; initial denaturation at 95°C for 2 min followed by 35 cycles of denaturation at 95°C for 30 s, annealing for 30 s, and extension at 72°C, followed by a final extension at 72°C for 5 min (Table 1). Products obtained from PCR reactions were run on 1.5% agarose gel stained with GelRed (Biotium) and amplicons of the correct size, relative to the positive control, were sent for Sanger sequencing (Eurofins Genomics, Louisville, Kentucky, USA).

**TABLE 1:** Primers and probes used for generating COI sequences and running qPCR and dPCR assays for optimization and developing the multiplex assay with corresponding critical PCR parameters.

Species	Oligo Name	Strand	Sequence (5′ ➔3′)	Annealing	Extension
Universal	LCO1490	Sense	GGTCAACAAATCATAAAGATATTGG	40°C	1 min 30 s
	HCO2198	Anti‐sense	TAAACTTCAGGGTGACCAAAAAATCA	40°C	1 min 30 s
*Boa constrictor* (BC)	BC_COIF	Sense	CCTGCCTAAGCATCCTTA	58°C	1 min
	BC_COIR	Anti‐sense	CTAGGACGTTGAAGATCTG	58°C	1 min
	BC_probe	Sense	CGAATGGAACTAACACAGCCCG	58°C	1 min
*Epicrates cenchria* (RB)	RB_COIF	Sense	TCACCACATGCATCAATA	58°C	1 min
	RB_COIR	Anti‐sense	GCAGTGATTATAACAGATCAG	58°C	1 min
	RB_probe	Anti‐sense	AACCAGCCTCAATACCAATATTTAACA	58°C	1 min
*Python sebae* (NAP)	NAP_COIF	Sense	TCCCACGAATAAATAACATAAG	58°C	1 min
	NAP_COIR	Anti‐sense	CCAGCTTCTACGTATGAA	58°C	1 min
	NAP_probe	Sense	CGCTACTCCTCCTCCTGTCTT	58°C	1 min
*Python bivittatus* (BP)	BP_COIF	Sense	CCACTATCAGGCAATATG	58°C	1 min
	BP_COIR	Anti‐sense	CAGCTAAGTGTAGTGAGA	58°C	1 min
	BP_probe	Sense	CCACTCAGGCCCATCAGTAGATC	58°C	1 min

**FIGURE 2:**
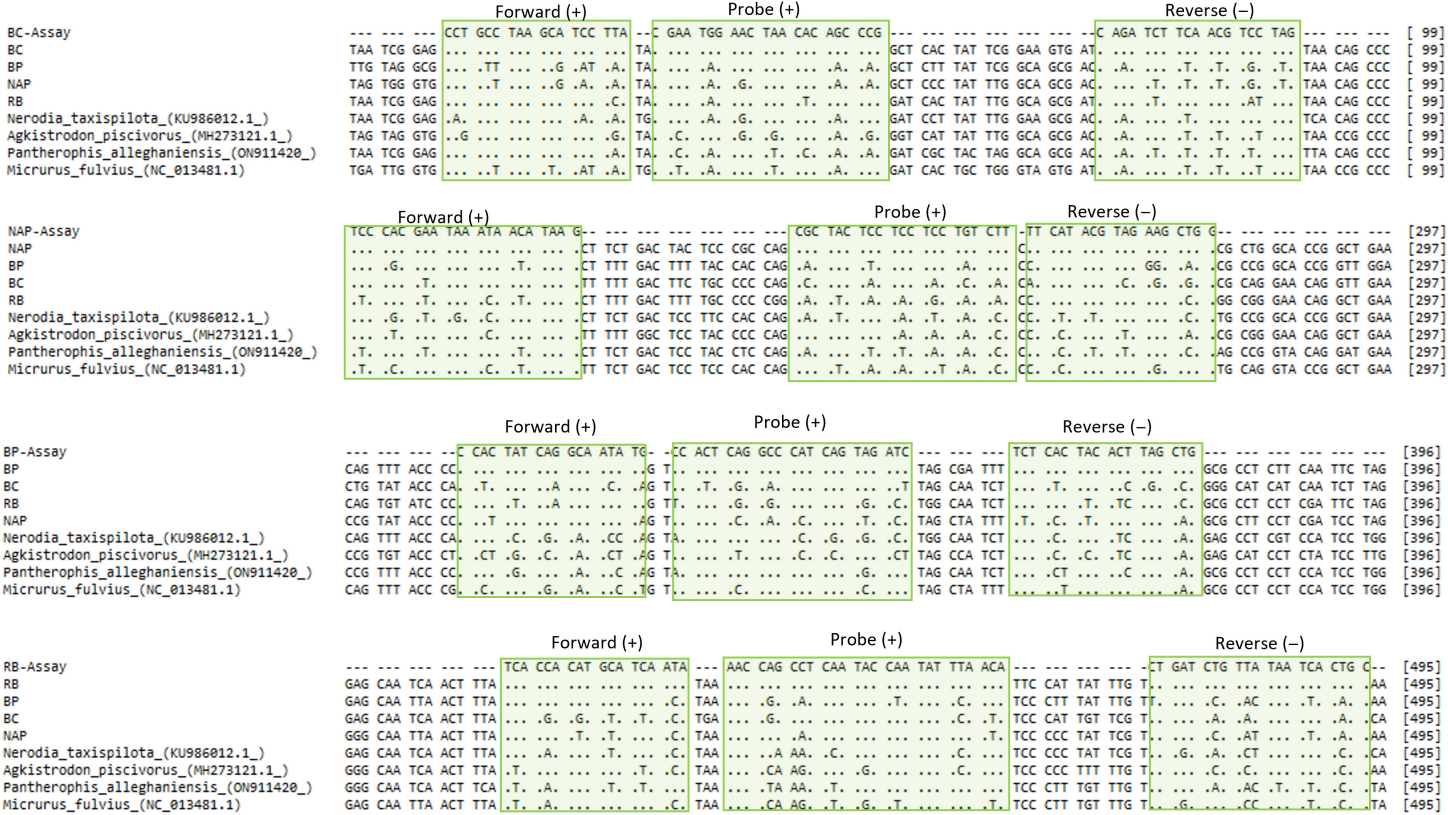
Target region of the COI gene used to design the multiplex assay aligned with corresponding assays on top rows compared to target species included in this study and some native species of snake commonly found in Florida to highlight variability present at primer and probe binding sites (green); number in brackets indicates the location within the 621 bp sequence obtained for target species using the LCO1490/HCO2198 primers; +/− indicate sense of the highlighted oligonucleotide; BC, *Boa constrictor*; NAP, North African python, BP, Burmese python and RB, rainbow boa.

Sequence data were assembled using DNA Baser (Version 4.36) (Heracle BioSoft SRL, Pitesti, Romania) and aligned using Clustal*W* as part of the MEGA7 package (Kumar, Stecher, and Tamura 2016). In addition to the invasive snake species used in this study, COI data available in GenBank for some common, native snake species in Florida (*Agkistrodon piscivorus*, *Micrurus fulvius*, *Nerodia taxispilota*, and *Pantherophis alleghaniensis*) were included in the alignment to verify assays encompassed regions with SNPs from possible environmental contaminant species and ensure cross‐amplification was not occurring with nontarget snake species (Figure 2). Sections of 100 bps displaying variability among the four species included in this study were selected and uploaded to OligoArchitect Online (Sigma‐Aldrich) using the “Dual‐Labeled Probe” tab. Assays were selected based on no predicted secondary structures, hairpins, primer dimers and an overall quality rating of 80 or higher. Each resulting assay was subsequently purchased as a TaqMan MGB (minor grove binder) probe with a 5′ FAM label and 3′ nonfluorescent quencher (NFQ) for optimization along with corresponding primers. Each assay was screened against its corresponding snake species with species‐specific primers only by standard PCR using a gradient to establish optimal annealing temperatures for the assay (based on the presence of a single, clearly defined band). For the gradient PCR, reactions were performed using the same concentrations as the initial PCR assays listed above with a gradient of 50°C to 60°C (at increments of 1.25°C). Each resultant assay was labeled according to abbreviated common names of the snake species for ease of labeling and presentation; *B. constrictor* specific assay = BC, *E. cenchria* specific assay = RB, *P. sebae* specific assay = NAP and *P. bivittatus* specific assay = BP.

### 2.3 qPCR and dPCR Optimization

All assays designed were subsequently screened against the original template used to generate sequences that resulted in the corresponding assays. Each assay specific to a snake species was screened in triplicate against template for the same snake species (representing positive controls) and screened in triplicate against the other three snake species template (negative controls) to ensure no cross‐amplification occurs. All assays were run on a QuantStudio 6 Flex qPCR system (Applied Biosystems, Thermo Fisher Scientific). Reactions were performed in volumes of 20 μL and comprised of 10 μL of TaqMan Universal Master Mix II with UNG, 10 μM for each oligonucleotide (forward primer, reverse primer, and probe), 10% polyvinylpyrrolidone (PVP‐40), 1 μL of DNA template with sterile dH_2_O added to reach final volume (20 μL). Thermal cycling conditions for qPCR assays were as follows; initial hold at 50°C for 2 min, initial denaturation at 95°C for 10 min followed by 35 cycles of denaturation at 95°C for 15 s and annealing/extension at 58°C (based on similar amplification success across species in standard PCR determined from gradient PCR) for 1 min.

Amplicons from the gradient PCR for each snake species were cloned using the pGEM‐T Easy Vector kit (Promega) following the manufacturer's instructions. The cloned vectors were then transformed into NEB Turbo Competent *E. coli* (New England BioLabs) and plated on Lysogeny broth (LB) plates containing 100 mg/mL of ampicillin (Alkali Scientific, Ft. Lauderdale, FL), 10 mg/mL X‐Gal and 8 mg/ML IPTG in solution (AG Scientific, San Diego, CA). Plates were incubated overnight and transformed colonies were screened for the clones with correct inserts using M13F/M13R primers. Clones with an insert of the correct size were incubated at 37°C overnight on a shaker at 250 rpm in 20 mL of LB broth containing 100 mg/mL of ampicillin (Alkali Scientific, Ft. Lauderdale, FL). Finally, plasmids were extracted using a QIAprep Spin Miniprep Kit (Qiagen) per the manufacturer's instructions and sent for Sanger sequencing (Eurofins Genomics) to confirm identity of the inserts. Plasmid eluate was subsequently diluted to 10^7^ copies/μL followed by a serial dilution to 10^1^ copies/μL.

Serially diluted plasmids for NAP were subsequently run with the corresponding assay on the QuantStudio Absolute Q Digital PCR System (dPCR; ThermoFisher Scientific) to establish optimal dilution concentrations. In addition, eluate from the raw tissue extraction protocol for NAP was diluted to 25 ng/μL, then subsequently serially diluted (10:1) three times and screened with the corresponding assay to determine optimal concentration for total DNA samples. Optimal concentration (50% to 10% positive partitions) was determined only using NAP because the same gene is being evaluated across taxa and similar qualitative metrics are present across assays, so with standardized concentrations for both samples and plasmids, optimal concentrations can be assumed to be consistent in other species. Optimal dilution factors were established to provide accurate florescence thresholds for scoring amplification results from eDNA samples (controlled or field samples).

### 2.4 Multiplex Optimization

The multiplex assay was purchased directly from ThermoFisher. The 5′‐ends of each species‐specific probe were labeled as follows: FAM‐NAP, VIC‐RB, ABY‐BP, and JUN‐BC. All assays tagged on 3′ end with MGB‐NFQ quencher.

Plasmid standards for corresponding species with appropriate insert and total DNA extract for each snake species at optimal concentrations (based on data from NAP dilution series for plasmids and total DNA) were screened using the multiplex assay. In addition, samples with optimal concentrations for each snake species were mixed in all possible combinations to reflect the possibility of an eDNA sample containing multiple targets.

All reactions were run in volumes of 9 μL and comprised of 1.8 μL Absolute Q Master Mix (5×), 0.45 μL dPCR assay (20×), 1 μL DNA template, and the remaining volume made up with UltraPure water. Thermal cycling conditions were as follows: 10 min initial denaturation at 95°C followed by 25 cycles of denaturation for 15 s at 95°C and annealing/extension for 1 min. at 58°C. All reactions were run on the QuantStudio Absolute Q Digital PCR System (ThermoFisher Scientific).

### 2.5 Limit of Detection for Known Quantities of DNA and Tissue

To determine the limits of detection with the dPCR system available, total DNA was extracted from Burmese python muscle tissue using the extraction protocol referenced above. The final eluate was diluted to 1 mL aliquots that had the following concentrations: 4 μg, 3 μg, 2 μg, 1 μg, 500 ng, 250 ng, 100 ng, 50 ng, and 1 ng. The 1 mL aliquots were added to 999 mL of distilled water then filtered through a 0.22 μm nitrocellulose membrane (Merck Millipore Ltd. Billerica, MA) using a sterile filter holder (Nalgene, Rochester, NY) and a vacuum pump (model no. 1HAB‐25‐M100X, Gast). The full liter of each sample was processed, and the filters were dried for 5 min. Filters were stored at −20°C until DNA extraction. DNA was extracted from each filter using the SPINeasy DNA Kit for Water (MP Biomedicals LLC USA) according to the manufacturer's instructions. Extracted DNA was quantified using a NanoDrop Lite spectrophotometer (ThermoFisher Scientific). Any samples with a concentration above 1 ng/μL were diluted to 1 ng/μL with sterile dH2O to avoid potential oversaturation of the dPCR chips (100% positive wells).

Additionally, different amounts of Burmese python muscle tissue (0.5, 0.32, 0.20, 0.12, 0.07, and 0.02 g) were macerated, using a tissue homogenizer (Omni International, Kennesaw, GA) fitted with plastic disposable generator probes. Macerated tissues were added to 950 mL of distilled water and the final volume brought to 1 L with distilled water, representing mock eDNA samples with known amounts of tissue. Samples were filtered and processed the same as those above.

### 2.6 Controlled eDNA Testing on Water and Soil Samples

**FIGURE 3:**
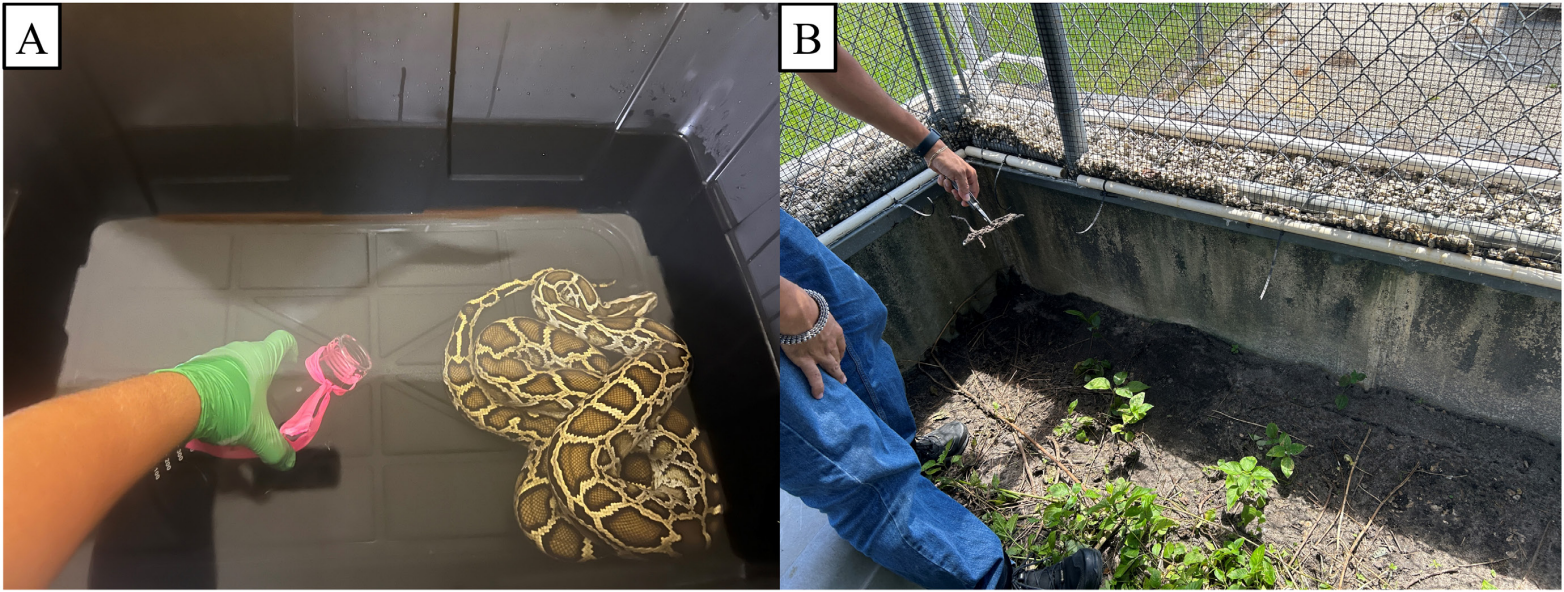
Control eDNA sampling: (A) water samples containing Burmese python and (B) soil/terrestrial sampling in enclosure occupied by a Burmese python.

Controlled eDNA water samples were generated by placing a live, female Burmese python (188 cm total length, 2.81 kg) in an opaque, black storage tote filled with tap water (Figure 3). Prior to filling with water and placing the snake within, the container was cleaned with a 10% bleach solution, rinsed with 70% ethanol and a final wash of distilled water to ensure sterile surface conditions. Autoclaved, glass Nalgene bottles were used to collect water samples of 1 L in volume. A baseline, negative control was collected prior to snake placement in water. After placing the snake in the water, 1 L samples were collected at time intervals of 5 min, 30 min, 1 h, 2 h, and 3 h. Water samples were stored in a cooler on ice and transported to the lab to be processed using the protocol outlined above.

Soil‐based eDNA samples were collected from an outdoor enclosure where a female Burmese python (292.8 cm total length, 12.3 kg) had been held for 8 days. Soil was collected by scraping a 16.5 cm stainless steel Scoopula (FisherScientific) along the ground where the snake had been observed resting prior to removal (Scoopula was completely filled with each scrape and 3 scrapes were collected for each time point) (Figure 3). Samples were collected at 24 h,1 week, and 2 weeks after the snake had been removed. Additionally, soil collected outside the enclosure (approximately 3 m away) served as a negative control. Soil was placed in 1 L glass Nalgene bottles and filled to 1 L with distilled water, mixed then processed using the protocol outlined above. Filters from both the water‐based and soil‐based eDNA samples had total DNA extracted and tested using the sample protocols as above with the previously developed multiplex assay. Additionally, dry filters were dabbed (until approximately 50% of the surface was covered in soil) in the same location as where soil scrapings were collected to compare sensitivity. Final eluate was diluted to 25 ng/μL.

## 3 Results

### 3.1 Assay Design and qPCR Optimization

The initial PCR reactions on all four snake species using the universal primer set resulted in positive amplification. For each species, a 621 bp product was obtained. Sequences for each species were deposited in GenBank; *B. constrictor* = PP556870, *E. cenchria* = PP556871, *P. sebae* = PP556869 and *P. bivittatus* = PP556868 (Table 1). Resulting primers and probes for each respective snake species are presented in Table 1 along with optimal annealing temperatures and times. All four assays were found to run optimally at 58°C for 1 min based on the gradient PCR (presence of single, well‐defined band, data not shown). When run by qPCR with the TaqMan probe included, all assays were successful in amplifying all three replicates of the target species without cross‐amplification of nontarget species (Table 2).

**TABLE 2:** qPCR results for evaluating potential of cross‐amplification across all four snake species for each species‐specific assay; cycle threshold values (Ct) = average of triplicates, No Ct = no amplification.

	Assay			
	*Boa constrictor* (BC)	*Epicrates cenchria* (RB)	*Python sebae* (NAP)	*Python bivittatus* (BP)
Species	Avg. Ct ± SE	Avg. Ct ± SE	Avg. Ct ± SE	Avg. Ct ± SE
*Boa constrictor*	19.29 ± 0.04	No Ct	No Ct	No Ct
*Epicrates cenchria*	No Ct	18.50 ± 0.44	No Ct	No Ct
*Python sebae*	No Ct	No Ct	18.58 ± 0.09	No Ct
*Python bivittatus*	No Ct	No Ct	No Ct	16.30 ± 0.11

### 3.2 dPCR Assay Validation and Optimization for Concentration Levels and Thresholds

Serially diluted plasmids for NAP were successfully amplified at all concentrations using the FAM‐labeled probe. Digital PCR chips were fully saturated (100% positive wells) at concentrations of 10^7^ copies/μL to 10^4^ copies/μL. At the 10^3^ copies/μL concentration, approximately 69% of the wells displayed successful amplification, with 10% and 1% successful amplification observed for 10^2^ and 10^1^ copies/μL concentrations, respectively. The optimal concentration for the NAP plasmids was between 10^2^ and 10^1^ copies/μL, having 95% confidence intervals (CI) values of 10% and below (Table 3).

**TABLE 3:** dPCR results for plasmid serial dilutions and sample dilutions for *Python sebae* (NAP) using FAM‐labeled probes.

Concentration	FAM‐NAP			
	% (+) Wells	Total wells	Conc. cp/μL	95% CI
Plasmid				
10^7^ copies/μL	100.00	20,473	22,978.85	4117.12
10^6^ copies/μL	99.96	20,450	17,890.08	1449.88
10^5^ copies/μL	99.98	20,469	19,769.38	2143.00
10^4^ copies/μL	99.96	20,446	18,162.27	1535.00
10^3^ copies/μL	68.80	20,345	2696.42	46.83
10^2^ copies/μL	9.62	20,345	234.11	10.15
10^1^ copies/μL	0.91	20,427	21.06	2.83
Sample				
25 ng/μL	99.94	20,455	17,039.43	1212.64
2.5 ng/μL	99.99	19,806	21,297.67	2978.09
0.25 ng/μL	32.26	20,317	901.56	21.70
0.025 ng/μL	3.15	20,395	74.16	5.52

The final eluate from the total DNA extractions of NAP also had successful amplification for all concentrations. At concentrations of total DNA of 25 and 2.5 ng/μL, chips were fully saturated (100% of wells reading as positive), while concentrations of 0.25 and 0.025 ng/μL total DNA yielded approximately 31% and 3% positive wells. The calculated copy number for concentrations of 0.25 and 0.025 ng/μL were 901.56 (21.7 95% CI) and 74.16 (5.52 95% CI), respectively (Table 3).

### 3.3 Multiplex Validation and Optimization

**FIGURE 4:**
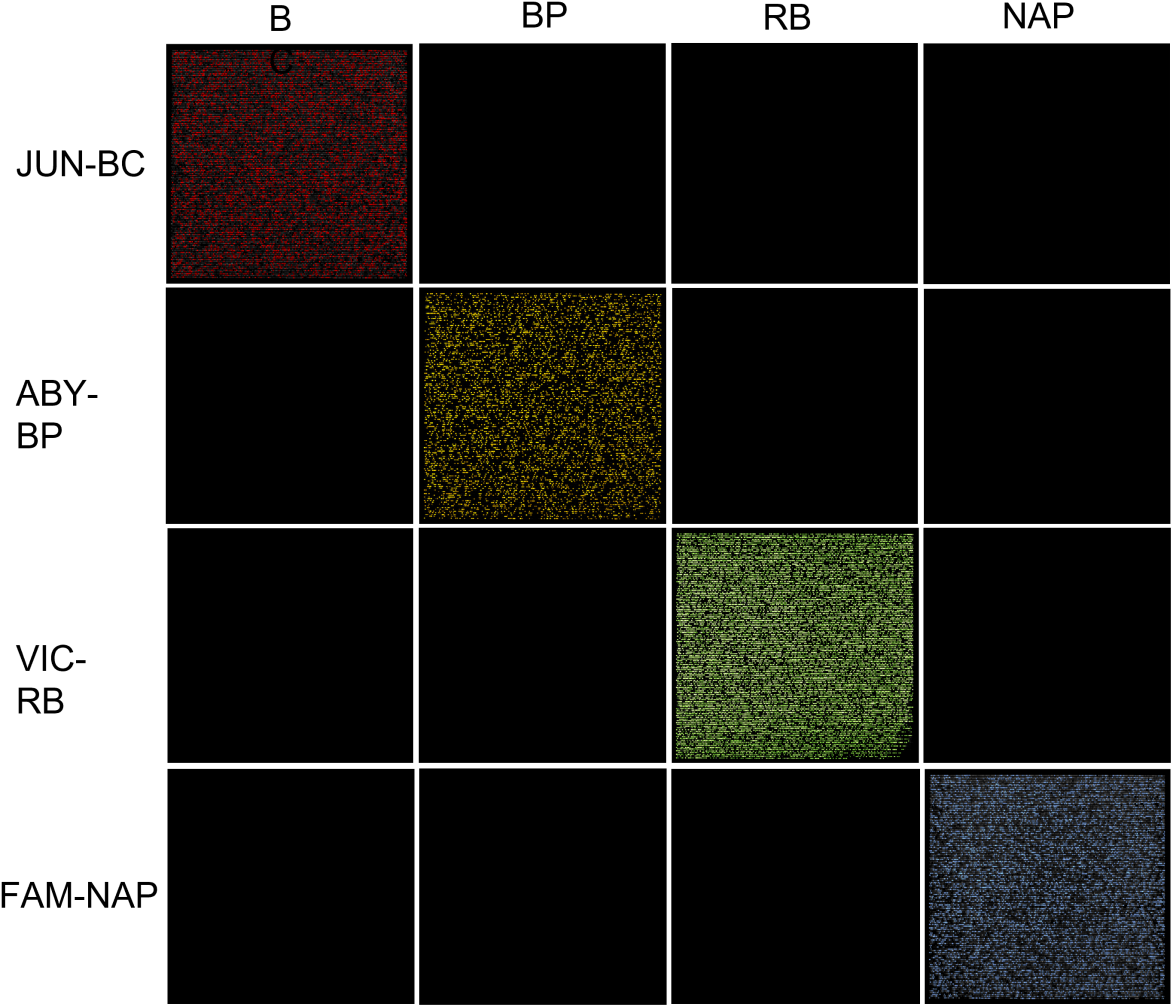
Chip images for the multiplex assay when screened against all four snake species (*x*‐axis) with reporters/probes (*y*‐axis) showing positive amplification for their corresponding species; red pixels = positive JUN florescence, yellow pixels = positive ABY florescence, green pixels = positive VIC florescence, blue pixels = positive FAM florescence, black pixels = no florescence/negative wells.

The multiplex assay successfully amplified for all reported dyes/assays for the corresponding snake species with no cross‐amplification across snake species detected (Figure 4). For the plasmids at 10^2^ copies/μL for all snake species, the range of positive wells was 9.6% to 37.6%, within optimal concentrations for estimating concentrations (Table 4). Total DNA extract diluted to 0.25 ng/μL for all snake species have percent positive partitions ranging from 11.29% to 33.26% positive partitions (Table 4). Florescence thresholds established for BC, NAP, RB, and BP are 128.615, 61.813, 453.251, and 655.377, respectively.

**TABLE 4:** dPCR results for the multiplex assay to validate function and verifying absence of cross‐amplification for the four snake species analyzed in this study based on optimal sample concentrations for plasmids and DNA extract.

	Plasmid (10^2^ copies/μL)				Sample (0.25 ng/μL)			
	JUN‐BC	ABY‐BP	FAM‐NAP	VIC‐RB	JUN‐BC	ABY‐BP	FAM‐NAP	VIC‐RB
BC								
Positives wells (%)	21.7	0.0	0.0	0.0	29.7	0.0	0.0	0.0
Total wells	20,364	20,364	20,364	20,364	20,386	20,386	20,386	20,386
Conc. cp/μL	566.11	0.0	0.0	0.0	814.66	0.0	0.0	0.0
95% CI	16.49	0.0	0.0	0.0	20.38	0.0	0.0	0.0
BP								
Positives wells (%)	0.0	9.6	0.0	0.0	0.0	31.41	0.0	0.0
Total wells	20,411	20,411	20,411	20,411	20,362	20,362	20,362	20,362
Conc. cp/μL	0.0	233.69	0.0	0.0	0.0	872.62	0.0	0.0
95% CI	0.0	10.12	0.0	0.0	0.0	21.25	0.0	0.0
NAP								
Positives wells (%)	0.0	0.0	18.1	0.0	0.0	0.0	33.26	0.0
Total wells	20,417	20,417	20,417	20,417	20,417	20,417	20,417	20,417
Conc. cp/μL	0.0	0.0	463.38	0.0	0.0	0.0	936.08	0.0
95% CI	0.0	0.0	14.71	0.0	0.0	0.0	22.15	0.0
RB								
Positives wells (%)	0.0	0.0	0.0	37.6	0.0	0.0	0.0	11.29
Total wells	20,324	20,324	20,324	20,324	20,411	20,411	20,411	20,411
Conc. cp/μL	0.0	0.0	0.0	1091.35	0.0	0.0	0.0	277.39
95% CI	0.0	0.0	0.0	24.42	0.0	0.0	0.0	11.10

Abbreviations: BC, *Boa constrictor* ; BP, Burmese Python; CI, confidence intervals; NAP, North African python; RB, Rainbow Boa.

**FIGURE 5:**
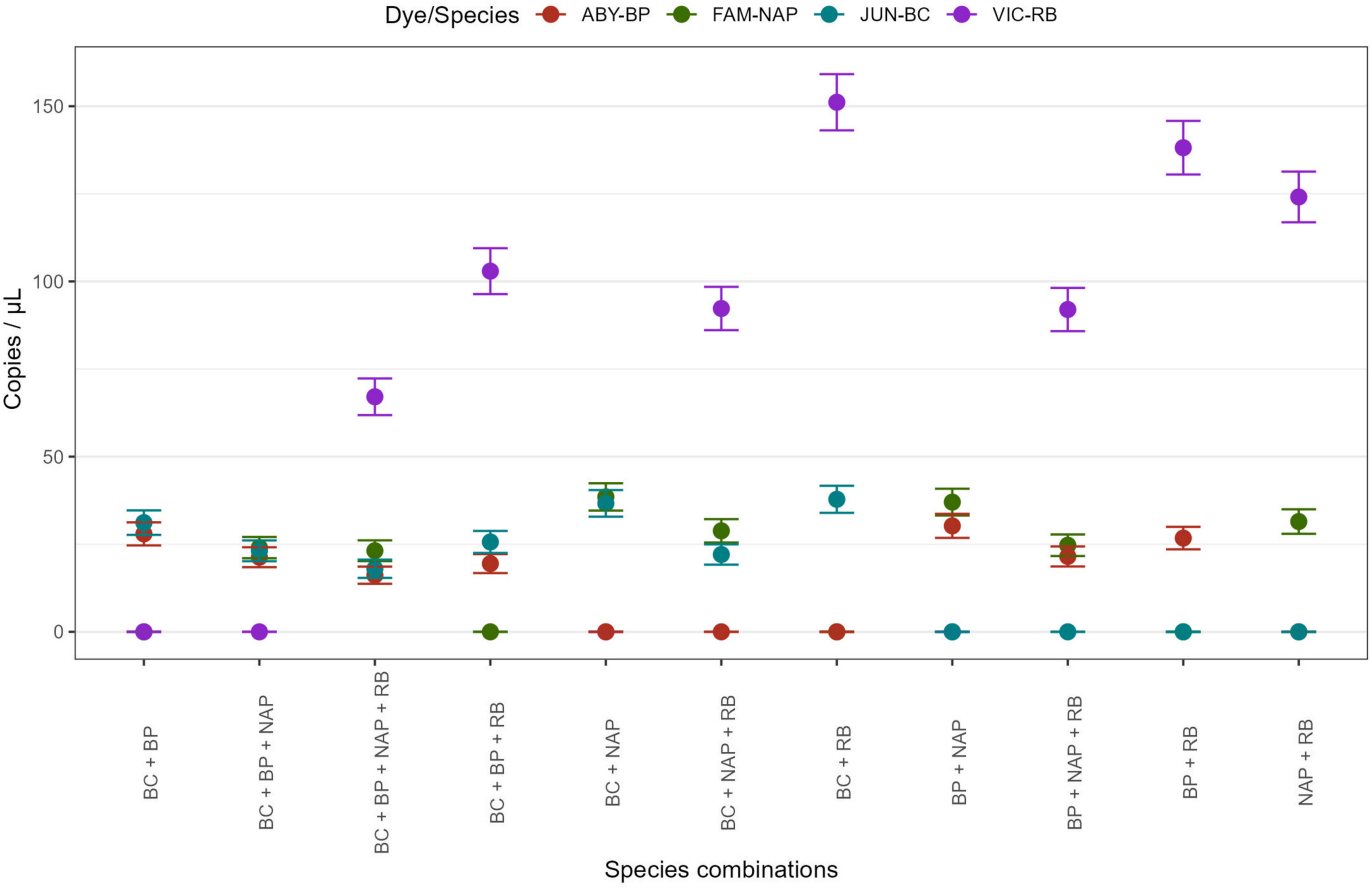
Absolute quantification of target DNA for each snake species when screened with tetraplex assay in mixed samples representing all possible combinations of multiple species present in a single sample.

All samples with mixed templates screened with the multiplex assay displayed positive/negative patterns that directly corresponded to which species were present in the sample (Figure 5). Generally, the difference in efficiency and copy number estimate did not significantly change among two‐species, three‐species, and four‐species mixtures. At low concentrations, the addition of additional template did not influence the assay. The 0.025 ng/μL concentration for RB, while yielding approximately 11 copies/μL by itself, consistently yielded significantly higher estimates in mixtures with other species. In addition, mixtures with RB template did display a reduction in copy number estimate as the amount of template increased, dropping from approximately 140 copies/μL in two‐species combinations to approximately 70 copies/μL when mixed with all three other species.

### 3.4 Limit of Detection for Known Quantities of DNA and Tissue

Raw DNA extracted from Burmese python muscle tissue and diluted to known concentrations in 1 mL aliquots placed within 999 mL of distilled water yielded positive dPCR results for all concentrations from 4 μg to 50 ng/L while the sample with 1 ng/L tested negative (Table 5). For all samples with macerated muscle tissue, positive detection was obtained with chips showing near 100% positive wells (Table 6).

**TABLE 5:** Extracted DNA from Burmese python muscle tissue in one milliliter (mL) eluate mixed into 999 mL of distilled water and quantified by dPCR after filtration.

Total DNA present	% (+) Wells	Total wells	Conc. cp/μL	95% CI
4 μg	61.79	20,409	2227.1	40.02, 40.756
3 μg	18.22	20,471	465.48	14.728, 15.209
2 μg	57.41	20,438	1975.96	36.507, 37.194
1 μg	36.36	20,449	1046.08	23.708, 24.258
500 ng	5	20,467	118.81	7.060, 7.506
200 ng	0.28	20,455	6.57	1.491, 1.929
100 ng	0.87	20,462	20.34	2.772, 3.209
50 ng	1.37	20,237	32.02	3.551, 3.994
1 ng	0.0	20,419	0.0	n/a

**TABLE 6:** Control eDNA samples of muscle tissue from Burmese python diluted to different concentrations quantified by dPCR after filtration.

Concentration	% (+) Wells	Total wells	Conc. cp/μL	95% CI
0.5 g/L^a^	99.99	19,975	22,921.96	4116.142, 5017.067
0.32 g/L^a^	99.51	20,467	12,318.05	444.380, 461.011
0.20 g/L^a^	99.86	20,462	15,264.17	833.228, 881.337
0.12 g/L^a^	99.09	20,351	10,868.38	326.154, 336.244
0.07 g/L	99.99	20,468	21,373.77	2978.872, 3461.207
0.02 g/L	99.94	20,466	17,225.96	1260.8211, 360.3093

^a^ Tissue macerate clogged filter so only 0.5 L was filtered and processed.

### 3.5 Controlled eDNA Testing on Water and Soil Samples

Water‐based eDNA samples tested by dPCR yielded positive results in all three replicates (Table 7). Baseline controls were all negative (no amplification observed) and in all three replicates, detection of Burmese python DNA occurred at 5 min post exposure to the water, with concentration estimates ranging from 4.44 to 203.01 copies/μL (Table 7). The lowest concentration estimates among replicates was not consistent at any time point, however in replicates two and three, the highest concentration was recorded at the 5 min timepoint (Table 7). Peak concentrations were recorded at 30 min in replicate one (181.2 copies/μL), 5 min in replicate two (77.5 copies/μL), and 5 min in replicate three (203 copies/μL). The overall average concentration estimate across all time points did not differ significantly between replicates one and two (48.2 ± 27.3 and 40.5 ± 11.7 copies/μL, respectively), however the overall average concentration in replicate three was substantially higher (117.8 ± 27.1 copies/μL) (Table 7).

**TABLE 7:** Control eDNA water samples from tank containing living Burmese python.

Sample	% (+) Wells	Total wells	Conc. cp/μL	95% CI
Replicate 1				
Baseline control	0.0	20,335	0.0	n/a
5 min	0.19	20,341	4.44	1.197, 1.638
30 min	7.53	20,415	181.19	8.838, 9.291
1 h	0.811	20,463	18.85	2.661, 3.98
2 h	1.03	20,433	23.91	3.025, 3.463
3 h	0.51	20,467	11.91	2.073, 2.510
Replicate 2				
Baseline control	0	20,469	0	n/a
5 min	3.29	20,467	77.51	5.637, 6.079
30 min	0.7	20,458	16.35	2.464, 2.901
1 h	2.25	20,407	52.66	4.604, 5.045
2 h	1.52	20,411	35.43	3.732, 4.172
3 h	2.58	20,459	60.41	4.944, 5.384
Replicate 3				
Baseline control	0	20,460	0	n/a
5 min	8.4	20,437	203.01	9.385, 9.840
30 min	5.85	20,445	139.66	7.693, 8.141
1 h	4.84	20,468	114.88	6.935, 7.381
2 h	4.15	20,448	98.16	6.386, 6.831
3 h	6.16	20,472	147.16	7.904,8.353

Soil‐based eDNA samples tested by dPCR showed positive results from the soil scraping at 24 h post‐removal (Table 8), giving a concentration estimate of 93.7 copies/μL. Extraction from the filter dabbed in the same area as the soil sample also tested positive, giving a concentration estimate of 13.05 copies/μL. At 1‐week post‐removal, python DNA was detectable, giving a concentration value of 48.71 copies/μL (Table 8) and at 2‐week post‐removal, python DNA was detectable with a concentration estimate of 12.25 copies/μL (Table 8). All negative controls (soil and filters) away from where snake was observed tested negative (Table 8).

**TABLE 8:** Control eDNA terrestrial samples taken from enclosure containing a living Burmese python.

Sample	% (+) Wells	Total wells	Conc. cp/μL	95% CI
Soil—corner 24 h	4.0	20,329	93.65	6.247, 6.694
Soil—corner 1 week	2.1	20,458	48.71	4.413, 4.853
Soil—corner 2 week	0.53	20,455	12.25	2.106, 2.544
Filter—corner	0.6	20,459	13.05	2.180, 2.617
Soil—negative control	0.0	20,398	0.0	n/a
Positive control	9.62	20,345	234.11	10.15
Water control	0.0	20,459	0.0	n/a

## 4 Discussion

The development of a multiplex dPCR assay that allows for the detection of four invasive species of snake in Florida is a critical advancement in the ability to analyze eDNA and will significantly strengthen early detection and monitoring efforts in the Florida Everglades. Previous studies have utilized dPCR for analyzing eDNA samples in Florida to monitor for pythons (Hunter et al. 2015, 2019; Orzechowski et al. 2019). However, the methods used were limited to the detection of only one species (in that instance *P. bivittatus*). With the current Absolute Q dPCR system, used in this study, a total of four reporter dyes are available, allowing for the development of more robust multiplex assays, and with the system being automated, the workflow for setting up, running, and analyzing samples is significantly reduced. To the authors' knowledge, this represents the first tetraplex dPCR assay for snakes. Other systems that have reported utility multiplex dPCR include salmonid pathogens (von Ammon et al. 2022), breast and ovarian cancer screening (Oscorbin et al. 2019), lung cancer screening (Oscorbin et al. 2022), and GMOs (genetically modified organisms) detection in food crops (Dobnik et al. 2016). Furthermore, the utility and value of tetraplex assays based on standard and quantitative PCR (non‐dPCR) have been previously demonstrated and include food allergens (Köppel et al. 2010), eDNA of food animals (Safdar et al. 2014; Hossain et al. 2017; Kaltenbrunner, Hochegger, and Cichna‐Markl 2018), drug‐resistant fungus (Arastehfar et al. 2018), paternity cases (Seidl, Jäger, and Seifried 1996), and influenza viral types (Henritzi et al. 2019). The utilization of this technology in areas such as human medicine, especially regarding cancer, and large‐scale agricultural monitoring highlight the power of this technology and validate the need to adapt this tool to invasive species monitoring where critical ecosystems are at stake. The development of a reliable and time efficient tetraplex dPCR is a necessary tool for wildlife monitoring as the rate of habitat encroachment by humans in Florida is exponentially increasing with Florida having the highest net migration rate from July 2021 to July 2022.

Cross‐amplification was not observed for any of the species analyzed in this study despite the close phylogenetic relationship among constrictor species examined. As invasive constrictor snakes in the families Pythonidae and Boidae share more genetic similarity compared to other families (Colubridae, Elapidae, and Viperidae) of native snakes found within Florida (Burbrink et al. 2020), we do not anticipate issues of cross‐amplification with native snakes in our assay. Sequence data on common native species found in Florida (Figure 2) highlight high numbers of SNPs across the assay region. Given that the assays did not cross‐amplify with the snakes screened in this study, cross‐amplification with other species with higher numbers of SNPs will likely not occur. The lowest number of SNPs between an assay in this study and nontarget species was the NAP‐assay and corresponding region for the Burmese python (both being in the same genus) with nine SNPs distributed across the assay binding sites. Convergent sequence variability has resulted in the same number of SNPs between the BC‐assay and corresponding region for *N. taxispilota*, however, as demonstrated by the data in this study, cross‐amplification will fail with this number of SNPs. Furthermore, reactions are run at 25 cycles to further ensure cross‐amplification does not occur. When an assay is properly designed and optimized with dPCR, a single target is detectable at 25 cycles. Any SNPs reduce efficiency of the reaction, meaning any cross‐amplification that would occur would begin appearing at cycles above 30. Previous dPCR assays, while designed to avoid cross‐amplification, were not evaluated against closely related taxa.

Preliminary screening of control eDNA samples in this study provides useful insights into the suitability of this technology for field monitoring. The raw DNA extract at different concentrations demonstrated little DNA loss in the filtration process and tissue macerate (more representative of an eDNA sample) filtered through demonstrated high yield in the workflow presented. The limit of detection with this dPCR system was approximately 50 ng of DNA in 1 L of water. At 1 ng/L, amplification was unsuccessful. The 50 ng/L sample gave estimates of 32.02 copies/μL so it is possible that concentrations between 50 and 1 ng/L may have yielded positive results, however time and resource constraints did not allow for further testing. Future efforts will focus on using blood diluted in water samples to address the physical limitations of measuring out such small amounts of tissue mass. Both water and soil eDNA controls had target DNA present in levels well above the limit of detection established in most cases. Some water samples and the week two post removal soil test gave estimates below the concentrations estimated for 50 ng/L, further suggesting that the lowest point for detection is between 50 ng and 1 ng/L of target DNA. These data demonstrate that the assay and protocol outlined herein are capable of detecting the target organism DNA when it is present in the environment and also displays a high level of sensitivity. While these data are intended to provide confidence in the technology and protocol to analyze eDNA samples, the success of its utility in the field needs to be evaluated and will be the focus of future research. Field variables such as distance from organism, persistence/rates of degradation under various conditions, effect of environmental contamination (inhibitors) on dPCR success can be investigated, but regardless, systematic sampling in time and space will be critical for successfully gathering and interpreting data for management programs. Finally, these data provide some useful insights into the persistence of eDNA in soil samples. Python DNA was detectable at 2 weeks post‐removal. It remains unknown whether the consistent drop in concentration over time was due to degradation, removal of substrate, and thus, eDNA or a combination of both. However, this serves as a useful metric for designing future experiments to thoroughly evaluate the effect of environmental factors on DNA degradation and an indicator that, in substrates that are semienclosed/protected, eDNA in soil is detectable for at least 2 weeks, and potentially longer.

Numerous factors are responsible for the increased sensitivity and reliability of dPCR results over other protocols (standard PCR and quantitative PCR). The physical partitioning of a sample over approximately 20,000 wells functionally allows for replicating a sample 20,000 times where detection of a single target (gene representing a target species) is possible. Detection of single target copies or even low target concentrations is either not possible or inconsistent with standard and quantitative PCR whereas detection of these same targets is observed in dPCR (Bahder et al. 2018). Additionally, the same partitioning of a sample also significantly reduces the impact of environmental inhibitors (substances that can inhibit PCR amplification, and hence detection of eDNA) due to significant reduction/elimination of inhibitor concentration per reaction well (Rački et al. 2014; Sidstedt et al. 2017). This degree of sensitivity allows for the detection of trace amounts of DNA in the environment (either terrestrial or aquatic), which can provide an effective means for early detection monitoring efforts of target invasive species. This may be particularly useful for the detection of species that are cryptic, exist at low population densities, or when only one individual of a non‐native species is reported. Finally, the dPCR system used in this study is fully automated, significantly improving the workflow and reducing the time and cost that has been associated with the previous generation of chip‐based dPCR and droplet digital PCR systems.

**FIGURE 6:**
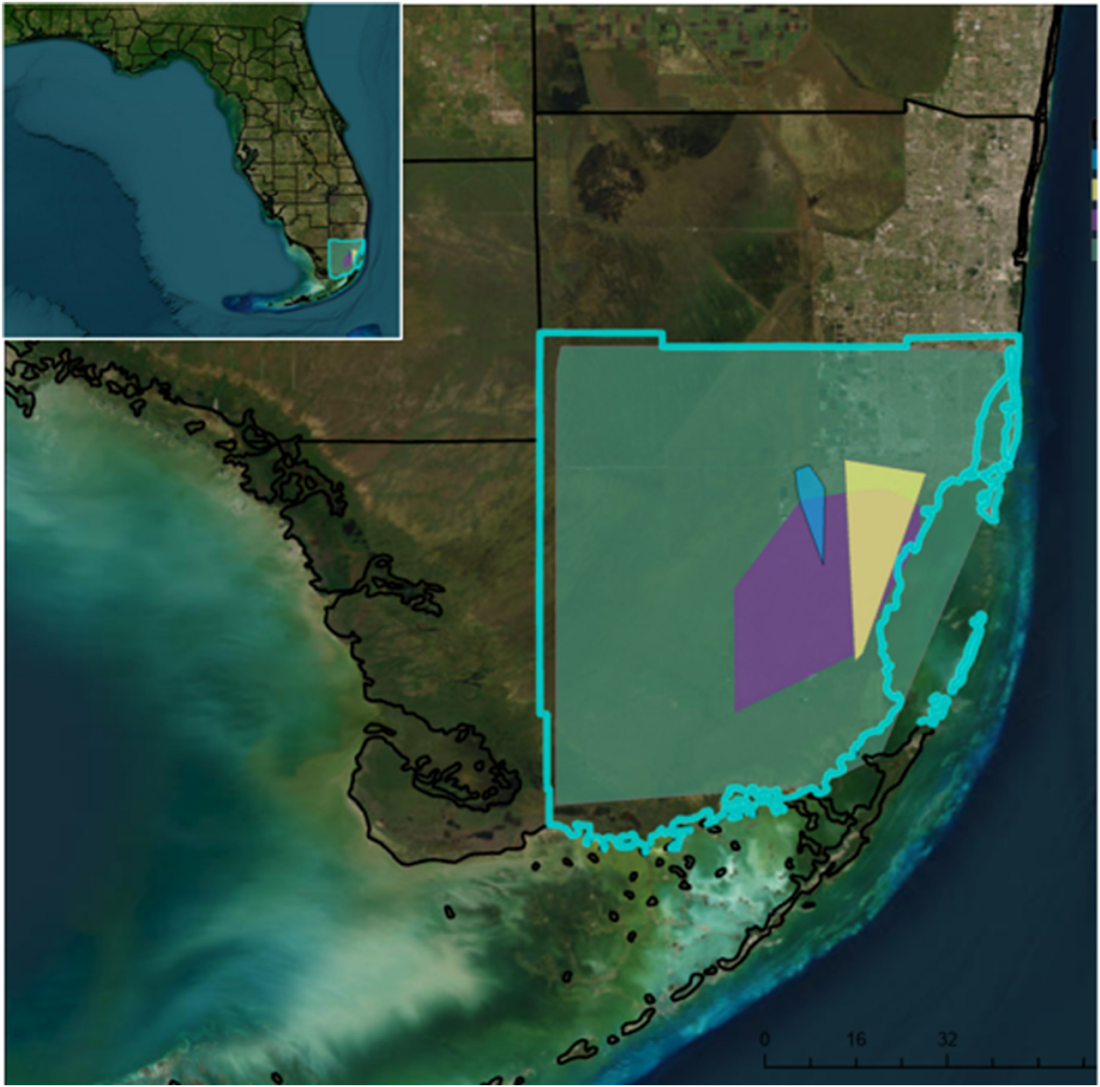
Established populations of Burmese pythons (green), northern African pythons (blue), boa constrictors (purple), and rainbow boas (yellow) are shown for Miami‐Dade County (blue outline) along with areas of range overlap among these species. Polygons representing each constrictor species were created using data available from EDDMapS (2024) using ArcGIS Pro, Version 3.2 and represent minimum bounding geometry Esri Inc (2023).

With the capability of multiplex testing, researchers and natural resource managers can reliably survey for multiple target species of interest simultaneously from an individual sample. This can reduce resources required for biosurveillance programs as well as increase the efficiency of monitoring programs in areas where numerous invasive species are present, which can enhance the ability to initiate an effective rapid response effort. Additionally, multiplex dPCR testing can be developed for any combination of target species, fitting the real‐time needs for the detection of invasive species across landscapes with multiple invasions. For instance, the range of Burmese pythons encompasses the distribution of northern African pythons, boa constrictors, and rainbow boas in Miami‐Dade County, FL (Figure 6) and having this tetraplex dPCR assay will allow for a more time and cost‐effective means to evaluate overlapping ranges and how (and if) these overlaps change over time. The capacity to test for multiple target species can also facilitate rapid and accurate confirmation of a species during EDRR efforts when reported sightings of non‐natives are ambiguous concerning species identification. Accurate species identification can aid managers in decisions regarding the appropriate response, or depending on the situation, whether to respond (i.e., a large constrictor is reported but the species identification is confirmed to be a Burmese python occurring in an area known to be teeming with pythons), which can inform prioritization of resources. Furthermore, sampling strategies can be developed in time and space that allow for the estimation of concentrations of target copy numbers to help managers determine if they are getting closer (or further) from a target organism or population.

In addition to early detection and monitoring efforts, multiplex dPCR testing may be an effective way to assess the outcome of removal efforts. For example, when post‐removal surveys for an invasive species are desired to demonstrate a decrease in occupancy or repeated zero detections (maximum control; Godfrey et al. 2023), use of dPCR can provide a reliable surveillance method for target species of interest that is accurate, rapid, and cost‐effective relative to traditional visual survey methods. This can be especially useful when species of interest for detection are cryptic or rare (i.e., snakes, crocodilians), and otherwise hard to detect using other methodologies. When management outcomes of interest are not based on repeated zero detections of a target species, but on the response of a native species impacted by an invader, multiplex testing may provide an additional advantage. For example, multiplex dPCR assays could be developed to assess the impact of python removal efforts on python occupancy across space and time, while simultaneously monitoring occupancy rates of mammals (i.e., opossums, racoons, and marsh rabbits) known to be impacted by pythons through predation (Dorcas et al. 2012; McCleery et al. 2015).

While utilizing multiplex dPCR assays for early detection and monitoring has advantages, these benefits are only advantageous if they are accessible to natural resource managers. As the number of invasive species continues to rise, the need for monitoring programs targeting multiple species increases. The use of dPCR assays can increase the efficacy of eDNA monitoring programs through the simultaneous monitoring of multiple target species. The dPCR assay developed herein for the detection of four invasive constrictor species increases the capacity for rapid and accurate detection of insipient populations and range expansions as well as long‐term monitoring for assessment of control efforts. Data gained from monitoring programs can aid natural resource managers in informed decision‐making concerning an appropriate response as well as allow managers to prioritize resources relative to the situation. For example, a manager may prioritize resources to respond to the detection of target invasive species near biologically sensitive areas. Additionally, with the ability to detect multiple target species in a sample and a reduced workflow for assays, multiplex dPCR may be a more cost‐effective means for implementing eDNA monitoring programs.

This study documents the successful development of a novel multiplex assay designed to detect eDNA of four species of invasive constrictors rapidly and accurately. Future work will assess multiplex testing under seminatural conditions with known presence of target species to validate results obtained in this study in the field, which will facilitate the development of a monitoring program for invasive constrictors in Florida. As Florida is a hotspot of biological invasions, additional multiplex assays targeting other established invasive taxa (i.e., lizards, fish, and crocodilians) negatively impact native ecosystems, as well as non‐native species with a high risk of adverse effects if they become established, are needed and should be a focal point of future studies to facilitate multispecies eDNA monitoring programs.

## Author Contributions

**Melissa A. Miller:** conceptualization (equal), funding acquisition (equal), project administration (equal), writing – original draft (equal), writing – review and editing (equal). **Melody Bloch:** data curation (lead), formal analysis (lead), methodology (lead), validation (lead), writing – original draft (equal). **Sergio A. Balaguera‐Reina:** conceptualization (supporting), formal analysis (equal), funding acquisition (equal), project administration (equal), writing – original draft (equal). **Kevin A. Olejniczak:** data curation (equal), investigation (equal), methodology (equal), writing – original draft (equal). **Cynthia A. Fussell Persaud:** data curation (equal), investigation (equal), methodology (equal), writing – original draft (equal). **Ericka E. Helmick:** data curation (equal), methodology (equal), resources (lead), software (lead), supervision (equal), writing – original draft (equal). **Frank J. Mazzotti:** conceptualization (equal), funding acquisition (equal), project administration (equal), resources (equal), supervision (equal). **Brian W. Bahder:** conceptualization (lead), formal analysis (equal), funding acquisition (lead), methodology (lead), project administration (equal), supervision (lead), writing – original draft (equal), writing – review and editing (equal).

## Conflicts of Interest

The authors declare no conflicts of interest.

## Benefit‐Sharing Statement

Benefits from this research accrue from the sharing of our data and results on public databases as described above.

## Data Availability

Molecular data generated in this study are available on GenBank and have been released (National Center for Biotechnology Information (https://www.ncbi.nlm.nih.gov/nuccore/)). Accession numbers for associated data are PP556870, PP556871, PP556869, and PP556868. All other data associated with the study is presented within the manuscript.
